# Respiratory rate as a X-ray-based biomarker for the longitudinal assessment of lung function and pathology

**DOI:** 10.3389/fmed.2025.1621104

**Published:** 2025-10-08

**Authors:** Kaveh Ahookhosh, Willy Gsell, Birger Tielemans, Greetje Vande Velde

**Affiliations:** ^1^Biomedical MRI, Department of Imaging and Pathology, KU Leuven, Leuven, Belgium; ^2^Institute of Mechanics, Materials, and Civil Engineering, UCLouvain, Louvain-la-Neuve, Belgium; ^3^Institute of Pathology, University Clinics Aachen, RWTH University of Aachen, Aachen, Germany

**Keywords:** respiratory rate, lung function, X-ray-based biomarker, pulmonary functional imaging, microCT

## Abstract

Respiratory rate (RR) is a valuable, yet underexploited lung functional parameter in preclinical lung research, aiding drug toxicity studies, lung disease assessments, stress, pain, and sleep research. It may also enhance translatability between animal and human studies. Longitudinal micro-computed tomography (microCT) lung data acquisitions not only contain spatial information on lung disease, volumes and patterns, but also temporal information covering many breathing cycles. This enables reliable and non-invasive extraction of lung morphological and functional biomarkers, including RR, with a single measurement from free-breathing animals, crucial for accurate measurements. Here, we aimed to develop a non-invasive pipeline, for longitudinal RR monitoring as a biomarker for lung function and pathology based on the X-ray projections of lung microCT acquisitions. First, we mechanically ventilated a mouse and scanned it using microCT at different breathing rates, 60 to 185 breaths per minute (bpm), serving as ground-truth data for our RR measurements. Next, we obtained raw intensity curves from these ground-truth X-ray projections, which contained noise and signals from multiple sources such as respiratory and cardiac cycles. To find the optimal algorithm and isolate the respiratory signals, we post-processed these raw intensity curves with different signal processing techniques. Adept at handling non-uniformly sampled signals in time domain, the Lomb-Scargle (LS) algorithm outperformed the other signal processing techniques, exhibiting robust prediction of RR with an error margin of 3%. Next, we applied this pipeline to benchmark the longitudinal RR data as a biomarker of lung damage and repair in a mouse model of lung epithelial injury. Our RR monitoring pipeline detected a transient loss of lung function in diseased mice, marked by a temporary RR decrease and a simultaneous increase in total lung and aerated lung volumes. Adopting this X-ray-based pipeline would allow lung researchers to non-invasively collect both morphological and functional data in a single measurement, improving insights into lung disease progression and host response thereto by providing relevant biomarkers. This approach contributes to facilitating translation of preclinical study results toward clinical trials.

## 1 Introduction

The lung is a complex organ that plays a crucial role in gas exchange. Lung diseases can have a profound effect on lung structure and function, resulting in the disruption of the gas exchange process (1, 2). To understand the pathophysiology behind lung diseases and evaluate therapeutic strategies, researchers rely on endpoint or longitudinal readouts of lung disease burden, host response and lung function in animal models (3, 4). While end-point measurements provide valuable information, they are static and cannot capture dynamic changes in lung disease progression. Therefore, developing methodologies for collecting reliable data on lung structure and function at multiple time points is essential for pathophysiology and therapy efficacy studies of which the study results translate efficiently toward the clinical context.

In preclinical lung research, microCT has increasingly been employed to qualitatively and quantitatively assess lung disease burdens over time in *in vivo* animal studies. This stems from its unique ability to provide imaging data with high spatial and temporal resolution, enabling accurate quantification of morphological and functional biomarkers (3–5). MicroCT has proven to be an effective tool for longitudinally assessing lung morphological changes in *in vivo* animal studies using rodent lung disease models. Morphological microCT-derived biomarkers such as total lung volume, mean lung density, aerated and non-aerated lung volumes have been effectively employed in *in vivo* longitudinal animal studies for assessing lung disease burdens (3, 4). While microCT is a robust tool for longitudinal lung structural assessments, its full potential in providing functional biomarkers remains to be explored. Plethysmography remains the primary method for lung function assessment in animal studies, either non-invasively measuring respiratory-related parameters with whole-body or head-out plethysmography, or invasively evaluating lung biomechanical properties with commercialized devices such as FlexiVent and Buxco (2). However, plethysmography methods, including both invasive and non-invasive techniques, treat the lung as a single unit, providing only global readouts that lack sensitivity to capture early regional lung damages. Combining lung anatomical and functional measurements into a single procedure reduces animal handling and distress, thereby improving accuracy, reducing experimental costs, and saving time. In response to this need, the emerging field of pulmonary functional imaging integrates anatomical and functional measurements within a single acquisition, utilizing imaging data at multiple time points to provide precise longitudinal and regional biomarkers of lung anatomy and physiology for animal studies.

Pulmonary functional imaging aims at innovating animal studies as well as patient diagnosis and prognosis, and includes various techniques, such as inhaled krypton and xenon dual-energy CT, inhaled gas hyperpolarized magnetic resonance imaging (MRI), and nuclear medicine methods using gaseous radionuclides, which have been used to regionally visualize and measure gas distribution, ventilation, perfusion, and gas exchange processes (2, 6). The input imaging data for pulmonary functional imaging should have high spatial and temporal resolution, enable quantitative measurements, and be obtainable non-invasively and longitudinally. These characteristics ensure the successful retrieval of spatially resolved data for regional assessment of lung morphometry and function, aiding in early diagnosis, monitoring, and treatment planning (6). Similarly, microCT, as the gold-standard imaging modality for preclinical lung research, provides all these features, making it an ideal data source for pulmonary functional imaging in the context of fundamental and applied studies in small-animal models of lung diseases. MicroCT-based pipelines for pulmonary functional imaging provide regional and longitudinal biomarkers, enhance the sensitivity of detecting the regional onset and progression of lung diseases, facilitate the development of new therapeutics, and enable the accurate assessment of treatment outcomes in preclinical lung research (7–11). These imaging-based pipelines may serve as an essential tool in addressing the translational challenge in the preclinical phase of developing new therapeutics for lung diseases, facilitating the effective translation of their benefits to clinical trials and ultimately improving patient care.

As respiratory-related lung functional parameters are crucial for diagnosing and monitoring lung diseases in clinical settings (12), their measurement is equally important in *in vivo* animal studies of lung diseases. Measurement of parameters related to lung volumes, such as tidal volume, has already been validated and established using 4D microCT data by capturing end-inspiratory and end-expiratory lung images in both rodents (7, 13–16) and patients (17–19). However, additional, reliable and validated tools for access to microCT-derived lung functional parameters related to breathing patterns are still lacking. Respiratory rate (RR) is a functional lung parameter associated with breathing patterns and is sensitive to various pathological lung conditions (20). RR has been utilized for decades in clinics to diagnose and monitor the progression of different lung diseases (21–25). Since Fieselmann et al.’s article in 1993, several other reports have shown that RR, as one of the four vital signs, is an important indicator of serious illnesses in intensive care unit patients, such as cardiac arrest and lung pathologies (26–29). For example, a RR ranging from 12 to 20 bpm is considered normal in human adults, while values over 25 bpm indicate exacerbation of chronic obstructive pulmonary diseases (COPDs) and interstitial lung diseases (ILDs) (30). In preclinical settings, longitudinal assessments of various rodent models for lung diseases, including bleomycin-induced pulmonary fibrosis (31), paraquat-induced lung injury (32) and naphthalene-induced lung injury (33) have demonstrated positive correlations between lung dysfunction and changes in RR. These studies underscore the sensitivity of RR in capturing lung damage and subsequent changes in lung function. The current state-of-the-art for longitudinal RR measurement in preclinical lung research on spontaneously breathing animals is limited to whole-body plethysmography, head-out plethysmography and camera-based methods, which lack spatial information of the lungs during disease progression (2, 34–36). This limitation in providing spatial information adds extra procedures to animal studies, complicating the experimental design. Additionally, these methods require animal handling, such as transferring the animals to an unfamiliar chamber or cage, even applying restraint in the case of head-out plethysmography, which may cause distress and alter natural breathing patterns. Therefore, a comprehensive, non-invasive method to longitudinally obtain morphological and functional data with a single measurement is still lacking.

Here, we propose a non-invasive X-ray-based pipeline for extracting the respiratory signal and monitoring RR at multiple time points in *in vivo* animal studies. We extracted raw intensity curves from X-ray projections, which include a superposition of signals from different sources, such as gantry rotation, cardiac movement, and breathing. To identify the most accurate signal processing technique for extracting respiratory signals from X-ray-based intensity curves, we evaluated the following methods: 1. Respiratory gating; 2. Decomposition techniques (including two different algorithms); 3. Fourier transform; 4. Lomb-Scargle (LS) algorithm. The accuracy of the signal processing techniques is first assessed by comparing the RRs obtained after signal processing of *ex vivo* mouse X-ray projections with ground-truth mouse ventilator-controlled RR input. Next, we applied the most accurate signal processing techniques to longitudinal *in vivo* X-ray projections acquired from a naphthalene-induced lung injury mouse model, to assess the validity of X-ray-based RR as a sensitive, functional biomarker of lung injury in a real-life application.

## 2 Methods and materials

### 2.1 Animals

All animal experiments were carried out in compliance with national and European regulations and were approved by the animal ethics committee of KU Leuven (P083/2023 and P236/2014). All mice, BALB/c and C57Bl/6J, were kept in a conventional animal facility with individually ventilated cages and free access to food and water.

### 2.2 Ventilator-controlled forced breathing

**FIGURE 1:**
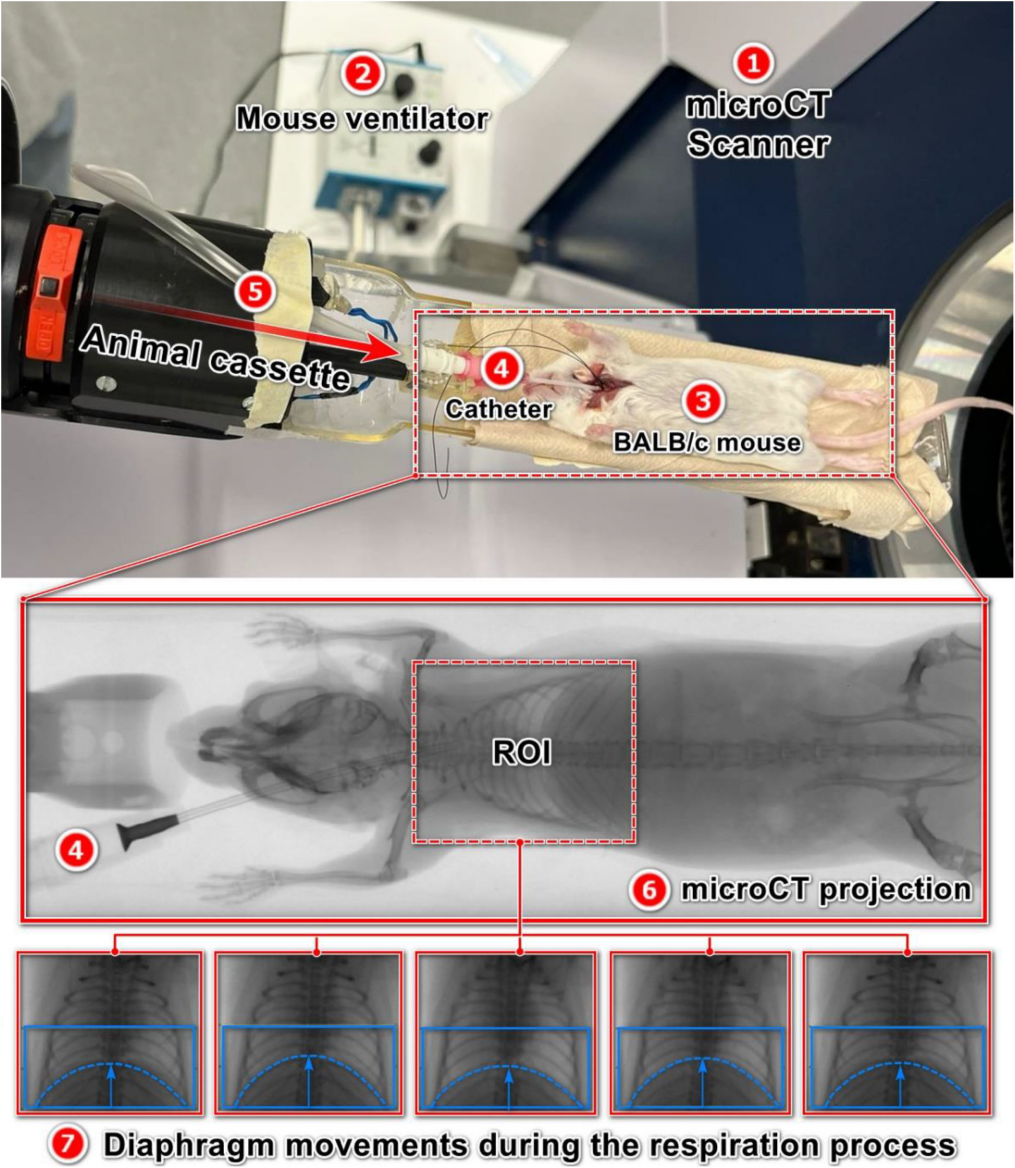
MicroCT **(1)** and experimental setup using a mouse ventilator: a mouse ventilator **(2)** is connected to the lungs of a deeply sedated BALB/c mouse **(3)** through a catheter **(4)** and a silicon tube **(5)**, attached to the trachea to control the respiratory rates during the microCT scanning. Diaphragm movements are captured through acquired X-ray projections **(6, 7)**. At each RR, the microCT scan repeated three times with the same setting.

An eight-week-old male BALB/c mouse was sedated through an intraperitoneal injection of 40 μl pentobarbital (150 mg/kg), followed by tracheotomy. A catheter was inserted into the trachea and secured with surgical suture to connect the mouse to a mouse ventilator (MiniVent, Harvard Apparatus) and ventilate the mouse during the microCT. The deeply sedated mouse was connected to the ventilator through a catheter to obtain ground-truth X-ray projections at different RRs (Figure 1), ranging from 60 to 185 bpm. The stroke volume was set to 200 μL during ventilation, based on the typical tidal volume of adult mice. At each RR, the microCT scan was repeated three times with the same setting to calculate the standard deviation of our RR measurements. During the scanning process, the mechanical breathing of the mouse was monitored using the physiological window provided by the scanner. Since the mouse was deeply anesthetized with pentobarbital, no signs of spontaneous breathing movements were observed.

### 2.3 Naphthalene-induced lung injury mouse model

In this animal study, two groups including eight male C57Bl/6J mice, each group with four mice, were employed (33). These mice were intraperitoneally injected with either naphthalene (NA) at a dose of 200 mg/kg body weight, dissolved in mineral oil (MO) as a vehicle, or they received only vehicle 10 mL/kg of mineral oil (MO) at the baseline. In this experiment, both the control and diseased mice were scanned with microCT at baseline, day-7, and 13 after inducing the lung injury.

### 2.4 MicroCT

MicroCT data were acquired with a dedicated *in vivo* microCT scanner with low radiation dose designed for longitudinal studies on small animals (SkyScan 1278, Bruker microCT, Kontich, Belgium). The system is equipped with a CMOS flat-panel X-ray detector with a native pixel size of 75 μm, providing high spatial resolution for a wide range of applications. The animals were anesthetized with isoflurane (1.5–2% in oxygen) for microCT at multiple time points. We used a time-resolved scanning protocol, where time is defined and recorded as the fourth dimension (4D), using the step-and-shoot scanning mode at 50 μm pixel size, obtaining 9 projections at each angular position known as list-mode acquisition (37, 38). The mouse was scanned in supine position using the following parameters: a 50 kV X-ray source voltage combined with a composite X-ray filter composed of 1 mm of aluminum, 350 μA current, 55 ms exposure time. The rotation step was set to 0.9°, covering a total angle of 220°. This scanning setting resulted in approximately 9 min of scan time for all the scans used in this study. The field of view for acquisition encompassed the entire mouse body, with a size of 10 cm. Reconstruction was confined to the lung area. This scanning protocol delivers 540–699 mGy per scan to the animal, which was shown by our group to be physiologically safe for longitudinal animal studies (39). The same microCT scanner and scanning settings were used to acquire imaging data from the mice in both the ventilator-controlled forced ventilation and naphthalene-induced lung injury experiments.

### 2.5 Imaging-derived breathing signal extraction

We obtained raw intensity curves based on the pixel intensity changes due to the diaphragm movements from the acquired 2D X-ray projection images. Thereto, the 2D X-ray projection images were imported into the DataViewer (version 1.5.6.2, Bruker) where several ROIs with benchmark angular positions separated by 45° rotation steps were manually placed on the lungs, encompassing the entire lung and diaphragm area (Figure 2.1). A simple rectangular-shaped ROI was used to cover the entire lung, as it was the fastest method to obtain a high-quality intensity curve.

**FIGURE 2:**
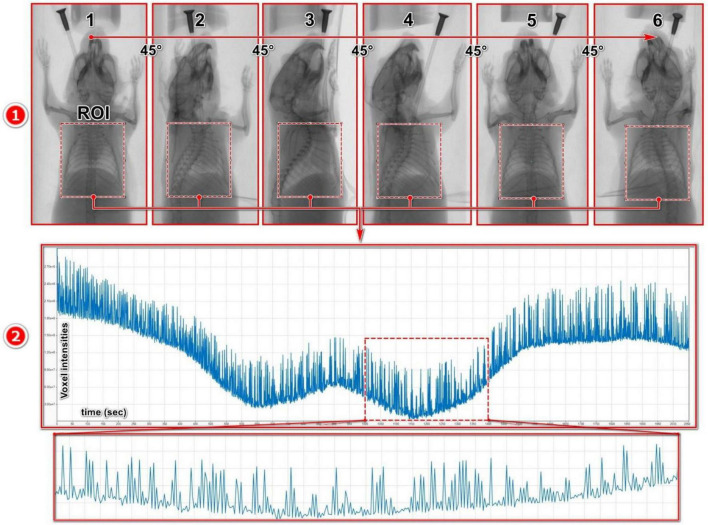
Breathing signal acquisition from projection images: **(1)** Multiple ROIs with benchmark angular positions separated by 45° rotation steps were manually placed on the lungs, encompassing the entire lung and diaphragm. **(2)** The exported intensity curve, derived from the signal acquisition process, represents the average pixel intensity within the ROIs across all X-ray projections within the associated timeframe.

For the angular positions between the benchmark positions, the ROI positions are linearly interpolated from the benchmark ROIs to save time and streamline the process. To maintain consistency throughout the analysis, the size of the ROIs was kept identical across all the projections. The raw intensity curves obtained from the projection images represent amplitude curves that depict the average pixel intensity (attenuation) changes within the ROI for each projection of the dataset. These intensity curves consist of a combination of signals induced by various sources, including gantry rotation, respiratory and cardiac cycles, as well as noise (Figure 2.2).

### 2.6 Signal processing techniques

#### 2.6.1 Built-in respiratory gating

**FIGURE 3:**
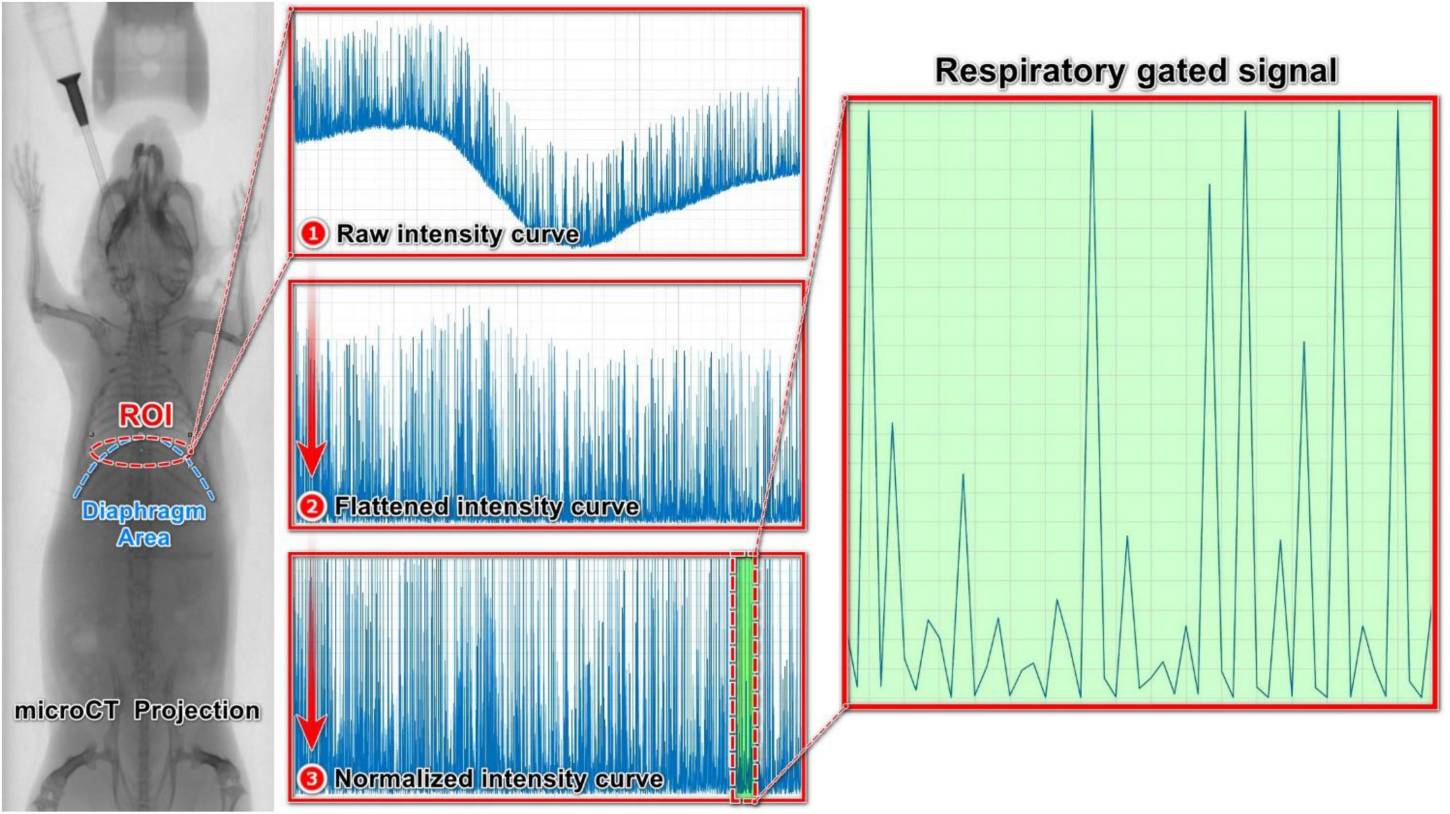
Extracting respiratory signal from a raw intensity curve using respiratory gating technique: **(1)** Raw intensity curve obtained from X-ray projections. **(2)** Flattened intensity curve, by subtracting the baseline of the raw intensity curve. **(3)** Normalized intensity curve, normalized by the local maximum of each angular position.

The first step in this technique is signal acquisition using an elliptical ROI that covers only the diaphragm area. The concept is to track diaphragm movements throughout the respiration process (38). Given the shape of the diaphragm, an elliptical ROI is better suited to capture the diaphragm movements accurately (Figure 3). We used the built-in respiratory gating in DataViewer for signal acquisition using 45° rotation steps between the benchmark ROIs. After the signal acquisition step with the elliptical ROI, we performed three additional steps to obtain a normalized curve for determining the respiratory signal. The initial step involved identifying the baseline of the intensity curve (Figure 3.1, raw intensity curve). This formation of the baseline curve is specific to intensity curves obtained from step-and-shoot scans. Since all projections were acquired at the same position for each angular step, the baseline intensity is determined by selecting the minimum intensity among these frames. The second step involved subtracting the baseline from the raw intensity signal to flatten the curve (Figure 3.2, flattened intensity curve). Finally, the flattened curve was normalized by the local maximum of each angular position (Figure 3.3, normalized intensity curve).

#### 2.6.2 Signal decomposition techniques

**FIGURE 4:**
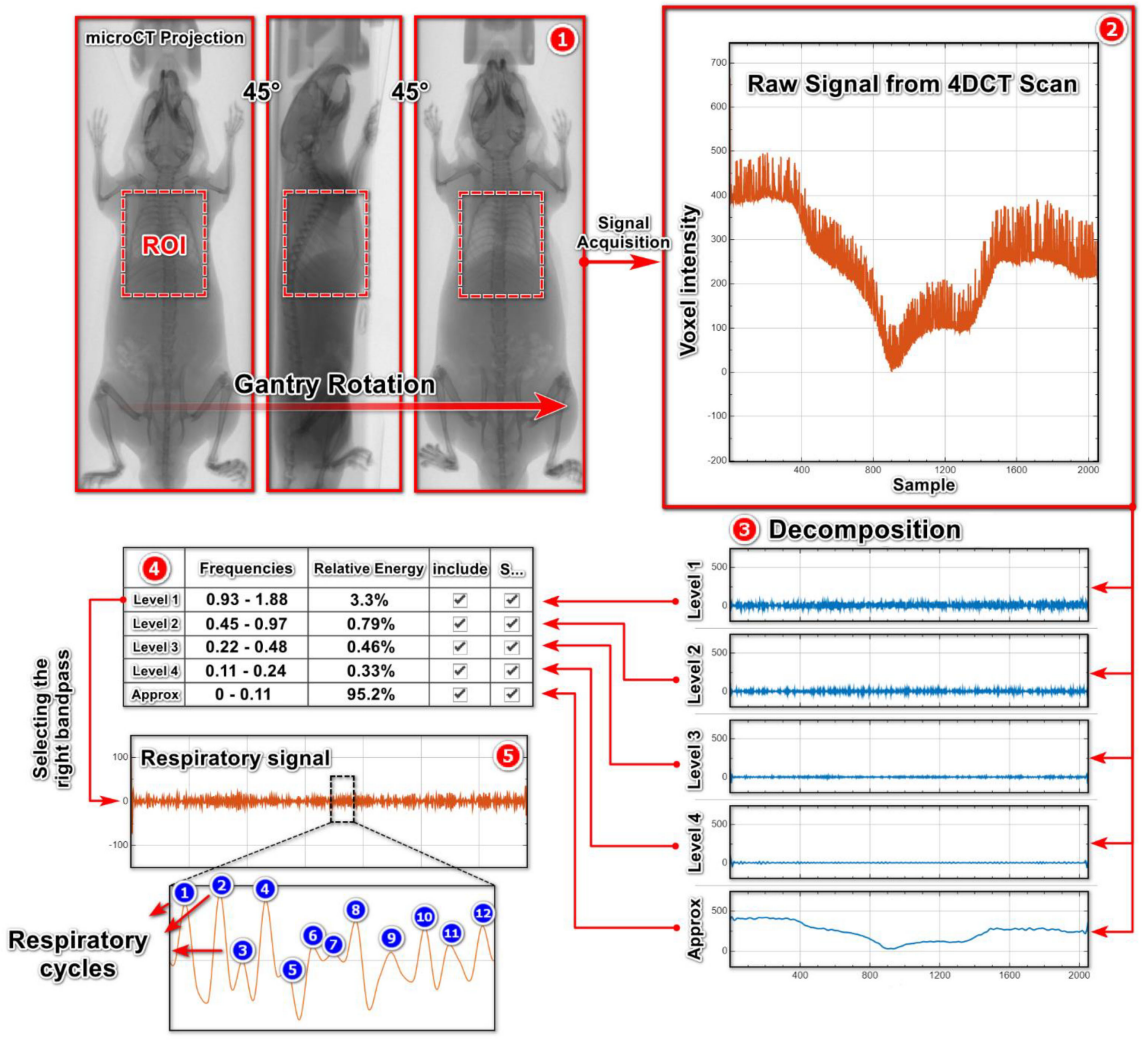
Stepwise extraction of respiratory signal from a raw intensity curve using MODWT and EMD algorithms: **(1, 2)** Signal acquisition from X-ray projections; **(3)** Decomposition of the raw intensity curve into different signal components; **(4, 5)** Selecting the right signal component based on its frequency range.

We employed two signal decomposition algorithms, MODWT and EMD, using the Signal Multiresolution Analyzer toolbox in Matlab. As the first step, we imported the raw intensity curve into the Matlab toolbox and associated the signal with the corresponding scanning time using the sample rate option. By employing either the MODWT or EMD algorithms, we decomposed the raw intensity curve into different signal decomposition levels that corresponded to distinct frequency and energy ranges of the acquired signal (Figures 4.3, 4). While MODWT is not adaptive to the content of the signal, EMD is signal-specific, and its decomposition is driven by the signal’s local extrema and oscillatory modes. The desired signal is extracted by selecting one of these signal components based on an additional information, such as the expected frequency range or energy characteristics of the respiratory signal, which is necessary to select the right signal component.

#### 2.6.3 FFT algorithm

**FIGURE 5:**
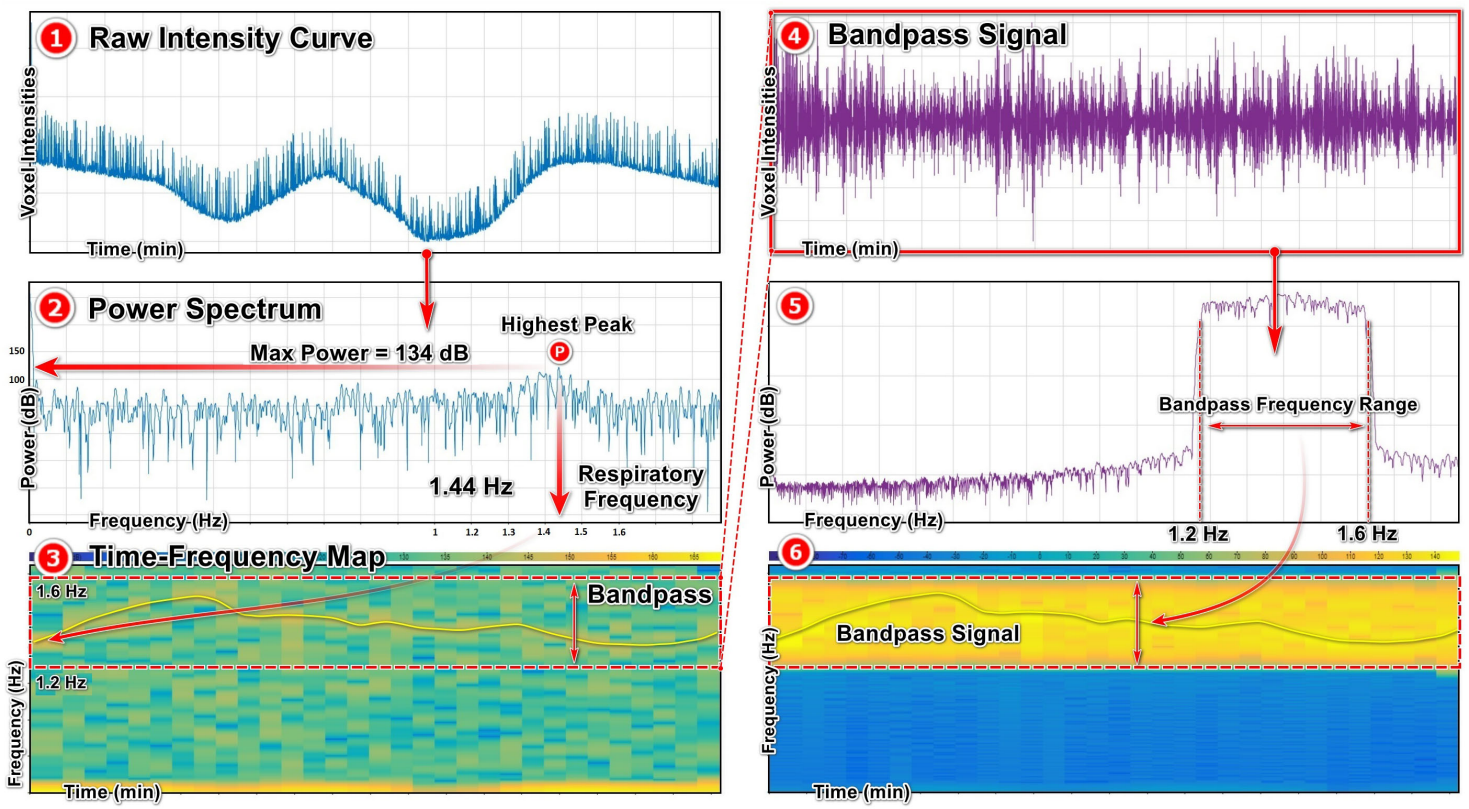
Extracting respiratory signal from a raw intensity curve using FFT algorithm: **(1)** Raw intensity signal obtained from X-ray projections. **(2)** Power spectrum map of the raw intensity curve using FFT algorithm, showing the highest peak with the maximum power and the corresponding frequency referring to the respiratory signal. **(3)** Time-frequency map of the raw signal, showing the most prominent signal inside the frequency range and the selected bandpass range. **(4, 5, 6)** The bandpass respiratory signal and the corresponding power spectrum and time-frequency maps.

We utilized the Signal Analyzer toolbox in MATLAB to extract respiratory signal from our raw intensity curves through determining the respiratory frequency with power spectrum density (PSD) analysis, which relies on FFT algorithm. The initial step is importing the raw intensity curve into the toolbox and associating it with the corresponding scanning time uniformly using the sample rate option (Figure 5). Subsequently, through power spectrum analysis, we identify the highest peak of the curve, indicating the dominant signal within the raw intensity curve. The peak with the highest power within the raw signal corresponds to the respiratory signal based on the selected ROI during signal acquisition, which provides us with the respiratory frequency represented on the *x*-axis. In this case with maximum power of 134 dB, the frequency is 1.44 Hz (Figure 5.2, Power Spectrum). In the time-frequency map, the dominant signal representing the respiratory signal is visible, allowing us to identify the appropriate bandpass range for signal extraction. Here, a bandpass filter between 1.2 and 1.6 Hz is the appropriate range for capturing the signal of interest with the frequency of 1.44 Hz (Figure 5.3, Time-Frequency map). Depending on the frequency of the respiratory signal for each mouse, the bandpass range for signal extraction was adapted in this study.

#### 2.6.4 Lomb-Scargle (LS) algorithm

**FIGURE 6:**
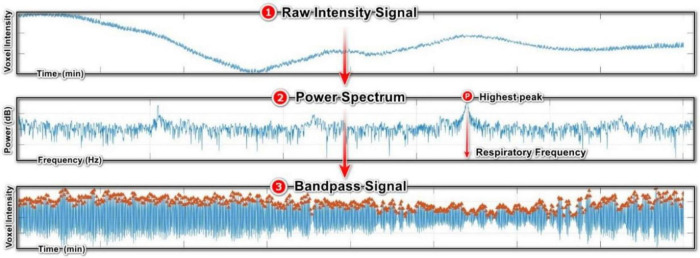
Extracting respiratory signal from a raw intensity curve using the LS algorithm: **(1)** Importing the raw intensity curve and associating it with the non-uniform time marks of the acquired pixel intensities. **(2)** Power spectral analysis of the raw signal using LS algorithm and identifying the respiratory frequency. **(3)** Bandpass the respiratory signal based on the respiratory frequency.

The same as FFT analysis, the LS method relies on PSD analysis, highlighting the respiratory signal with its highest peak and the corresponding frequency (Figure 6). To perform PSD analysis using the LS algorithm, we developed custom MATLAB code in-house. Here, by utilizing the identified respiratory frequency, we apply a bandpass filter to isolate the respiratory signal for further analysis and extract lung functional parameters.

### 2.7 Statistical analysis

The data were analyzed using GraphPad Prism (version 8.02, GraphPad Software Inc., San Diego, USA). Data are presented as mean ± standard deviation (SD). Normality was assessed using the Shapiro–Wilk test and visual inspection of QQ plots. A two-way repeated measures ANOVA with Sidak’s multiple comparison *post-hoc* test was performed to analyze the RR data from the Na-induced mouse model.

## 3 Results

In order to accurately measure RR as a biomarker of lung function, we aimed to develop a non-invasive pipeline based on microCT X-ray projections for *in vivo* longitudinal animal studies. As a first step, we set up an experiment to obtain ground-truth data for our RR measurements and ensure that we are measuring actual RRs from free-breathing mice using our signal processing pipeline. We connected a healthy mouse to a ventilator to regulate its RR during microCT across a wide range of RRs (60–185 bpm). In this experiment, only one eight-week-old male BALB/c mouse was used. However, by employing a wide range of RRs, the variability typically observed across a population of anesthetized mice, and the effects introduced by different anesthetics, including their duration and dosage, are effectively represented (40). The stroke volume during ventilation was selected based on the typical tidal volume of adult mice (41). As the scanning parameters were previously optimized by our group to ensure a radio-safe protocol for longitudinal studies of murine disease models (39), no modifications were made to these parameters in the current study. This radio-safe scanning protocol includes recording several projections per angular position, which helps improve image quality, enhance the signal-to-noise ratio, and reduce noise. When the primary objective is lung functional imaging, the number of projections per angular position can be reduced to a single acquisition, minimizing anesthesia time, scan duration, and X-ray dose. In this pipeline, since we were interested in high image quality to extract both morphological and functional biomarkers, we acquired multiple projections per angular position. On the ground-truth X-ray projections, we placed an ROI over the lung region from which obtained raw intensity curves containing signals from various sources, including respiratory and cardiac cycles, microCT gantry rotation, and noise (Figure 2). To extract the ventilator-induced respiratory signals from the raw intensity curves, we tested various signal processing techniques to find the most effective method for isolating them from noise and other unwanted signals. The respiratory signals extracted using different signal processing techniques were then used to measure RRs, and the accuracy of these techniques was evaluated by comparing them with the input-RR from the mouse ventilator.

**FIGURE 7:**
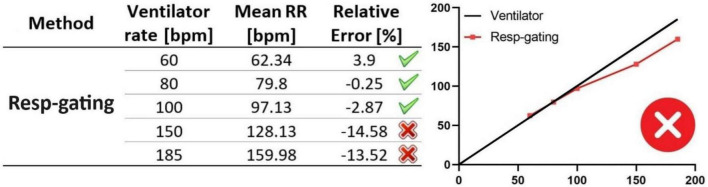
Evaluation of respiratory gating technique for RR monitoring using ground-truth RR values. In the table (left panel), RRs obtained from the respiratory gating technique (mean RRs, second column) are compared to those from the ventilator (ventilator rates, first column), considered the ground truth. RRs represent the mean of three measurements, with error bars indicating standard deviations, obtained from three datasets acquired under the same ventilator rate and microCT settings. The graph (right panel), shows the deviation of respiratory gating accuracy from the ground-truth RRs (ventilator rates). Respiratory gating demonstrates high accuracy for RR monitoring below 100 bpm, but it tends to underestimate those above this threshold.

The first signal processing method was the built-in respiratory gating technique in Dataviewer, least complex method that involves tracking diaphragm movements throughout the respiration process. Using this technique, RRs were obtained from the ground-truth X-ray projections and compared against the mouse ventilator RRs. The respiratory gating demonstrated a high level of accuracy in predicting the ground-truth RRs below 100 bpm, resulting in negligible differences with an error margin of 4% (Figure 7). However, as the RRs increased to 150 and 185 bpm, resulting in faster diaphragm movements within the physiological breathing frequency range of mice, the relative error between the ground-truth RRs and the output of the respiratory gating technique increased up to −14%. Therefore, we conclude that the respiratory gating technique, when applied to RRs above 100 bpm resulting in faster diaphragm movements, cannot track all respiratory cycles and tends to underestimate RRs to an unacceptable extent.

**FIGURE 8:**
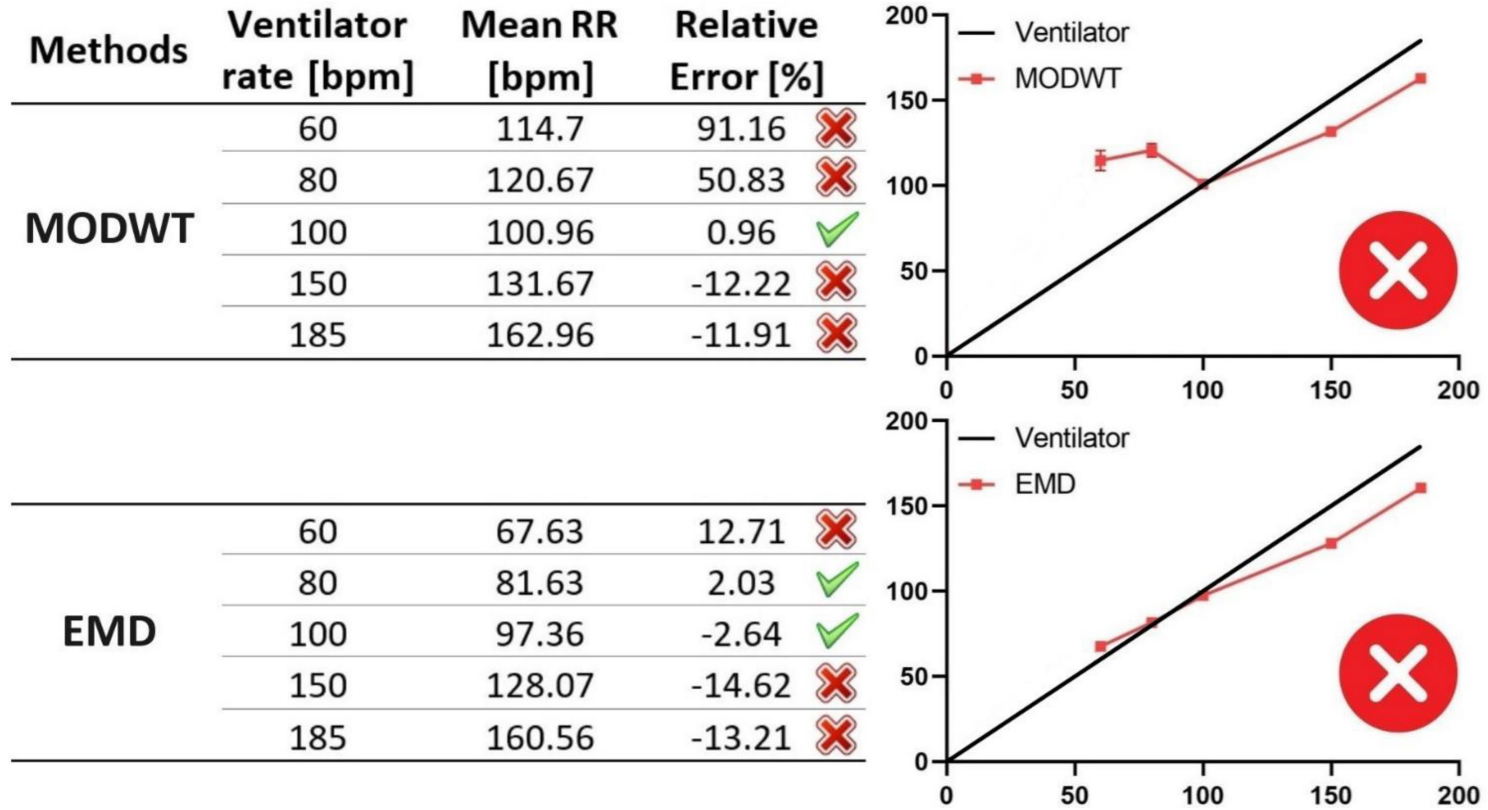
Assessing signal decomposition techniques for RR monitoring with ground-truth RR values. In the table (left panel), RRs obtained from the MODWT and EMD algorithms (mean RRs) are compared to those from the ventilator (ventilator rates), considered the ground truth. RRs represent the mean of three measurements, with error bars indicating standard deviations, obtained from three datasets acquired under the same ventilator rate and microCT settings. The accuracy of both methods fluctuates across the entire range of RRs, with EMD exhibiting better accuracy compared to MODWT. The graph (right panel) shows the deviation of MODWT and EMD accuracy from the ground-truth RRs (ventilator rates). MODWT and EMD algorithms are inaccurate and inconsistent in measuring the ground-truth RRs.

As we observed that the respiratory gating technique fails to capture all of the breathing cycles in RRs over 100 bpm and tends to underestimate the ground-truth RRs, we next tested two signal decomposition techniques, MODWT and EMD. We applied these two algorithms to scans of the ventilator-induced breathing experiment (ground-truth scans), decomposing the raw intensity signals into various components with different frequencies (Figure 4). Then, we extracted the respiratory signals based on the physiological breathing frequency range of mice that may be expected during lung disease experiments with lightly anesthetized animals. At 60 bpm, notable disparities become evident in the data produced by the signal decomposition algorithms compared to the ground-truth RRs, as measured by the relative errors (Figure 8). The MODWT significantly overestimated the ground-truth RR, with an approximate relative error of 91%. The EMD also overestimated the ventilator RR, but with a lower approximate relative error of 12%. Both algorithms exhibit higher accuracy toward 100 bpm, with an error margin of approximately 2%. However, their accuracy decreased once again as the RR approached 150 and 185 bpm (Figure 8). We conclude that The signal decomposition techniques demonstrated inconsistency in predicting the ground-truth RRs, i.e., overestimating of the RR below and above 100 bpm rendering them unreliable to predict the RR across the range of mouse breathing frequencies.

**FIGURE 9:**
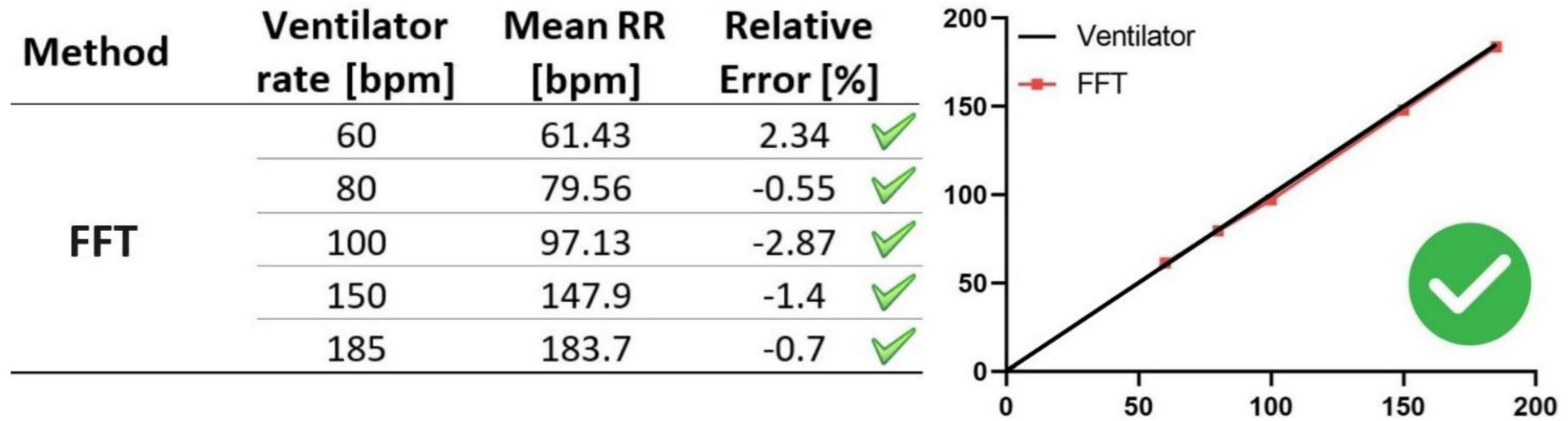
Evaluation of the FFT technique for RR monitoring with ground-truth RR values. In the table (left panel), RRs obtained from the FFT algorithm (mean RRs) are compared to those from the ventilator (ventilator rates), considered the ground truth. RRs represent the mean of three measurements, with error bars indicating standard deviations, obtained from three datasets acquired under the same ventilator rate and microCT settings. The graph (right panel) shows the deviation of FFT algorithm accuracy from the ground-truth RRs (ventilator rates). FFT algorithm exhibits accurately retrieves the ground-truth RRs across the entire range, with a minimal error margin of just 3%.

Due to the low accuracy and inconsistency of the signal decomposition techniques in predicting the ground-truth RRs, we assessed a fourth technique, the FFT algorithm, a computationally efficient method that offers a faster alternative to discrete Fourier transforms. We observed that The FFT algorithm demonstrated high accuracy in predicting the ground-truth RRs across the entire range of the ventilator RRs, with an error margin of only 3% (Figure 9). At 60 bpm, the FFT algorithm only slightly overestimates the ground-truth breathing frequency with relative error of approximately 2%, while at other RRs, this method tends to slightly underestimate the ground-truth RRs with the maximum relative error being around 3% at 100 bpm. We therefore conclude that the FFT algorithm can accurately extract respiratory signals from raw intensity curves and accurately measure RR.

**FIGURE 10:**
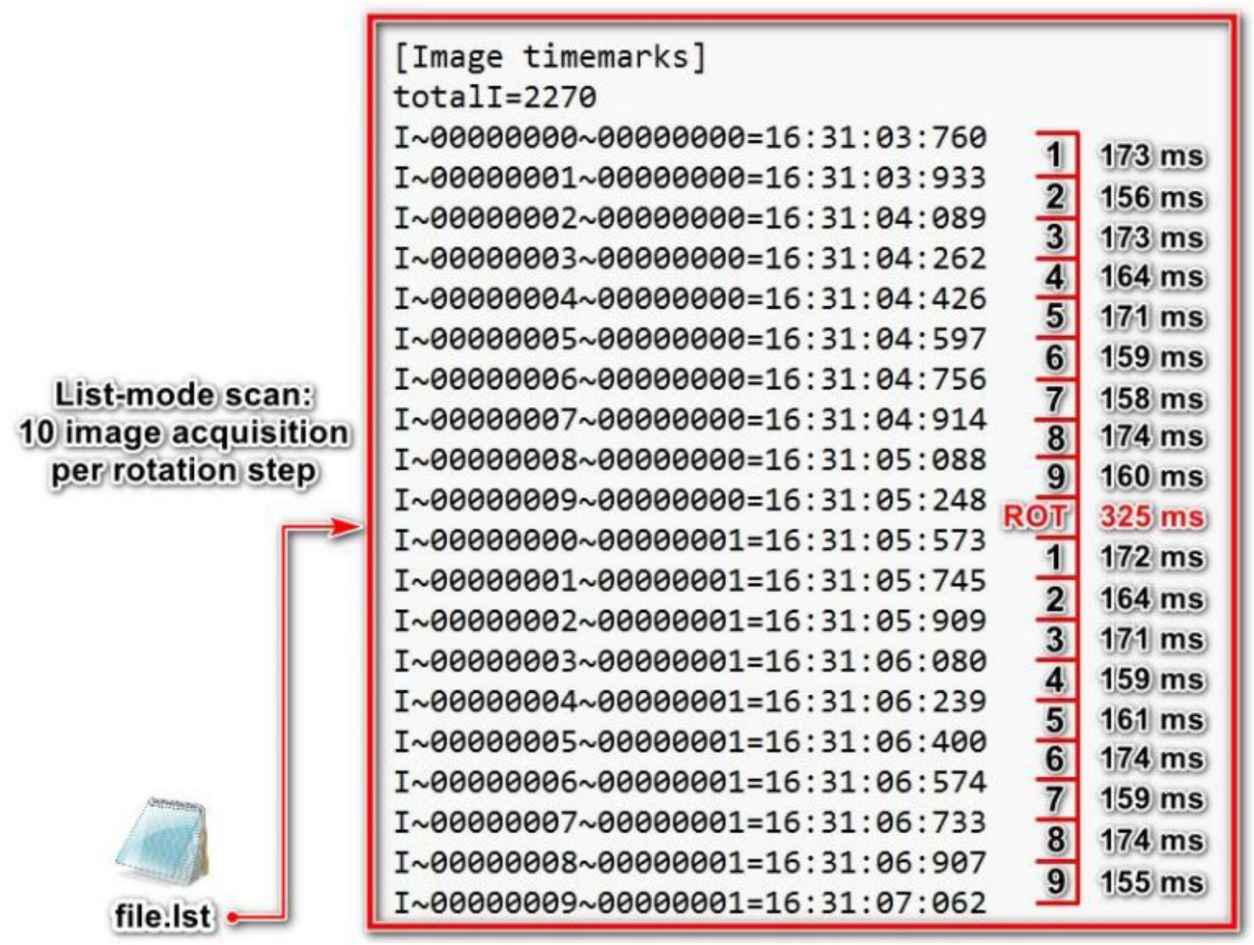
Time marks of the acquired X-ray projections in a list-mode scan, recorded and stored in a text file (file.lst). These image time marks show non-uniform time intervals between X-ray projections acquired in the step-and-shoot scanning mode. ROT here shows the rotation step between two angular steps during scanning.

Despite the accurate RR measurements using the FFT algorithm, its accuracy could be further improved. This is due to the fact that the FFT algorithm assumes that raw intensity curves are uniformly sampled in the time domain, whereas they are inherently non-uniform. The time intervals between the obtained X-ray projections vary due to the step-and-shoot scanning mode (Figure 10), resulting in intensity curves that are non-uniformly sampled in the time domain. Therefore, a signal processing technique capable of unmixing non-uniform signals could enhance the accuracy of RR measurements.

**FIGURE 11:**
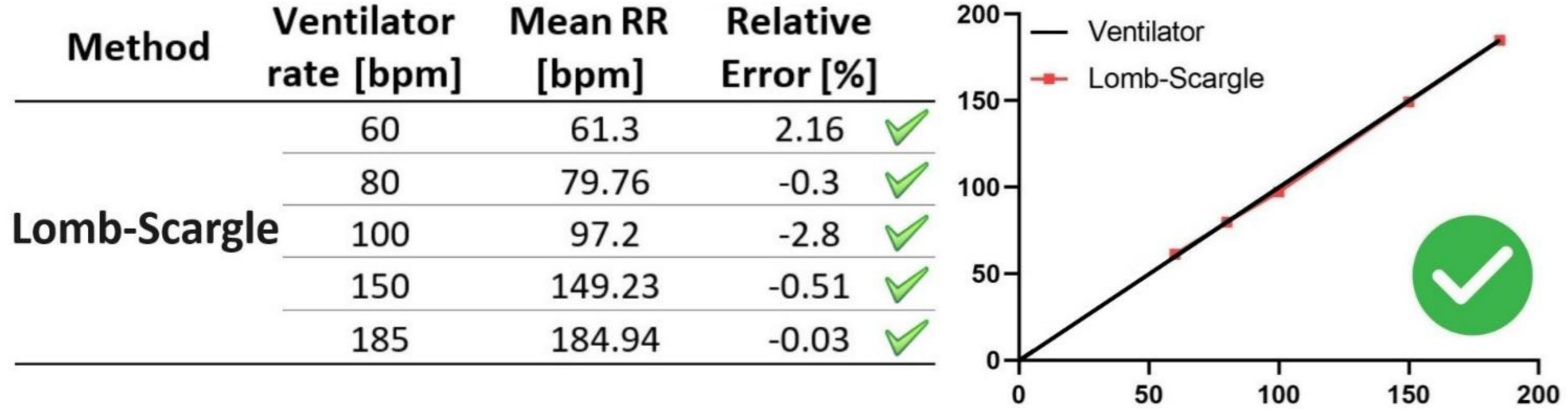
Evaluation of the LS algorithm for RR monitoring with ground-truth RR values. In the table (left panel), RRs obtained from the LS algorithm (mean RRs) are compared to those from the ventilator (ventilator rates), considered the ground truth. RRs represent the mean of three measurements, with error bars indicating standard deviations, obtained from three datasets acquired under the same ventilator rate and microCT settings. The accuracy of RR monitoring using the LS algorithm is improved across the whole range compared to the values obtained from the FFT algorithm. The graph (right panel) shows the deviation of LS algorithm accuracy from the ground-truth RRs (ventilator rates). The LS algorithm accurately and consistently predicted the ground-truth RRs.

Next, we utilized the LS algorithm, which is capable of handling signals that are unevenly sampled in the time domain or even have missing samples (42), to assess its ability to improve the accuracy of predictions for the ground-truth RRs. The LS algorithm retrieves very well the ventilator-induced RR over the entire range from the 60 to 185 bpm (Figure 11). The LS algorithm slightly improves the measured RR accuracy for all of the tested RRs, compared to the FFT. This highlights the low impact of assuming uniformly sampled intensity curves in the time domain on inducing inaccuracies in RR predictions. We conclude that the LS algorithm is most accurate to extract the RR from non-uniformly sampled intensity curves.

**FIGURE 12:**
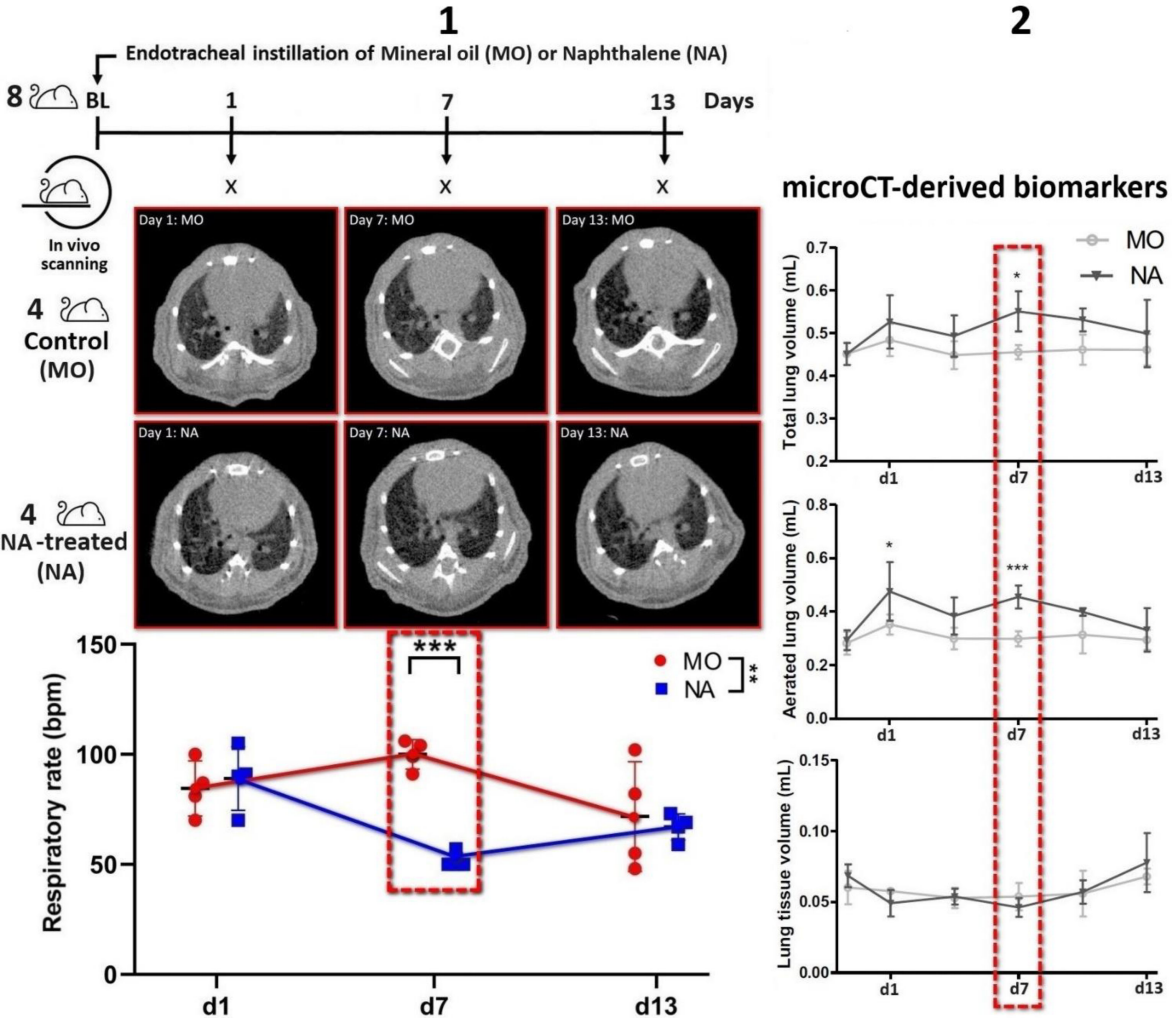
X-ray-based RR monitoring as a lung function biomarker in a naphthalene **(**NA**)**-induced lung injury mouse model (33). **(1)** From top to bottom: timeline of the experiment, microCT data of control and NA-treated mice at 100 z-positions after the first bifurcation of the airways, and corresponding measured RR values. Statistical analysis with two-way repeated measures ANOVA test shows a significant difference between the MO- and NA- groups on day 7, with a reduction in RR values for the NA-treated group. On day 13, RR values for the NA- group increase again, indicating the recovery of the mice 13 days after the NA injection. **(2)** MicroCT-derived biomarkers: Total, aerated, and lung tissue volumes extracted from the same microCT data. Adapted with permission from Springer Nature from (33), Copyright © 2018, Springer-Verlag GmbH Germany, part of Springer Nature, https://doi.org/10.1007/s00204-018-2161-8. Total and aerated lung volumes, aligned with RR values, show significant differences between the MO and NA groups on day 7, restoring back to baselines on day 13. Data are represented as mean ± SD. *p < 0.05, **p < 0.01, and ***p < 0.001 compared to MO as analyzed by two-way repeated measures ANOVA.

Next, to validate our X-ray-based pipeline for RR measurement using the LS algorithm, that we selected as the most effective signal processing technique, we employed the X-ray projections from a longitudinal micro-CT study on a NA-induced lung injury mouse model. This mouse model mimics lung airway epithelial damage and repair processes, making it ideal animal model for evaluating RR as a biomarker for the transient lung function impairment that was established in this model (33). NA- and vehicle-induced control mice were longitudinally scanned with microCT, and we extracted the RRs at multiple imaging time points from the X-ray projections of the two groups of mice (Figure 12). 1 day after induction, when lung injury has not yet set in, the RRs of both groups did not alter. However, on day 7, the RR significantly decreased in the NA-mice compared to the controls for the NA-treated mice. Subsequently, on day 13, RR values for the NA-treated mice returned to baseline, suggesting their recovery almost two weeks after the injection. These observations aligned with transient changes in microCT-derived biomarkers, i.e., total and aerated lung volumes that were elevated at day 7 (Figure 12), followed by restoring back to control values on day 13. These RR and volume changes happened while lung tissue volumes in NA-exposed mice remained unaltered, an indication of no overt signs of inflammation occurred. These data indicate that RR is a valid and sensitive biomarker of transient epithelial lung damage even when micro-CT scans do not show overt signs of inflammatory infiltration or signs of lung damage.

## 4 Discussion

In this study, we developed a non-invasive, X-ray-based pipeline for longitudinal RR monitoring as a lung function biomarker in *in vivo* animal studies. Our X-ray-based pipeline enables extraction of longitudinal RR data while simultaneously providing imaging data for measuring additional lung functional and anatomical biomarkers of lung disease burden and host response thereto, such as ventilation-related parameters, tidal volume, aerated and non-aerated lung volumes (3–5), all from a single acquisition. Measuring lung anatomical and functional biomarkers based on microCT reduces the need for additional procedures on animals, such as plethysmography techniques for lung function measurements, thereby reducing experimental complexity, costs and time. Furthermore, since the imaging protocol applies to free-breathing animals under anesthesia, it minimizes the impact of animal stress on the accuracy of the output data. Given that the scanning protocol involves anesthetizing animals, there is the impact of anesthesia on their breathing pattern to consider. Several studies evaluated the effects of anesthetics on respiratory variability in laboratory animals including mice (43, 44), rats (45), and rabbits (46). Isoflurane, the most commonly used anesthetic for experimental interventions in rodents in longitudinal animal studies, can induce respiratory and cardiovascular depression at concentrations exceeding 2% in oxygen, leading to hypoxemia, hypercapnia and hypotension. In contrast, a concentration of 1.5% isoflurane in oxygen has been shown to maintain stable respiratory and cardiovascular parameters (47, 48). In comparison to other anesthetics, isoflurane and sevoflurane, administered in oxygen at concentrations of 2.8% and 4.9%, respectively, caused respiratory depression with hypercapnia and acidosis in spontaneous breathing mice, marked by a decrease in RR from normal values of conscious mice, around 160 bpm, to values around 60 bpm (43). In a similar study, the effects of isoflurane (3% concentration in room air) and ketamine (a weight-based intraperitoneal dose of 100 mg/kg) on respiratory patterns of rats were monitored using whole-body plethysmography (45). Both anesthetics altered the breathing patterns of the rats, while isoflurane decreased RR, ketamine increased it, suggesting that different mechanisms initiated by each anesthetic influenced breathing patterns. Focusing on the effects of isoflurane concentration levels on the respiratory patterns of rodents, Kato et al. (49) examined the cardiorespiratory parameters of different strains of rats under increasing stepwise levels of isoflurane concentrations (1.5, 2, 3, 4, and 5%). RR values decreased in a concentration-dependent manner in all strains. However, at each concentration level, no significant differences in RR values were observed between rats of the same strain or between rats of different strains. Therefore, at a certain isoflurane concentration, a similar RR reduction can be expected in animals regardless of their group, ensuring that the effects of lung disease are not masked by isoflurane-induced respiratory depression. Confirming these results from the literature, our RR measurements in the NA-induced lung injury mouse model show that, despite potential respiratory depression induced by 1.5–2% isoflurane in oxygen in the imaging protocol, the distinction between groups due to NA-induced lung injury remains detectable.

By comparing multiple signal processing techniques using X-ray projections from a ventilator-controlled forced breathing experiment, we could identify the method that could most accurately unmix respiratory signals from X-ray-based intensity curves. The LS algorithm was the most effective in extracting respiratory signals, due to its capability of handling signals that are unevenly sampled in the time domain. This ability allows the LS algorithm to handle uneven breathing, including sighs, asymmetric breaths, and varying breathing rates over time in a more realistic way compared to other signal processing techniques. Moreover, the LS algorithm is the most robust to perform well for cases in which the X-ray projections are acquired non-uniformly throughout time. The X-ray projections used in this study were acquired using a 4D time-resolved scanning protocol, which records the exact time marks of each X-ray projection, operating in a step-and-shoot mode, where multiple projections are obtained at each angular step of the gantry rotation with the X-ray source. The rotation steps between the angular positions in this scanning mode cause non-uniform image intervals between X-ray projections. Consequently, the intensity curves obtained from these imaging data were non-uniformly sampled in the time domain, which contained noise and signals from different sources such as cardiac and respiratory cycles and gantry rotation of the microCT. To employ LS algorithm, an in-house code needed to be developed to isolate the desired signal from the raw signal. Once the code is in place, the process of extracting the respiratory signal was faster compared to the other signal processing techniques and the results are more accurate, especially for RRs close to 200 bpm. Therefore, the LS algorithm is the most accurate and optimal method for extracting respiratory signals and measuring RR with microCT data that provide exact time marks of the X-ray projections. Furthermore, by strategically positioning the ROI over the diaphragm and the tip of the heart during the raw intensity signal extraction, the LS algorithm can be employed to extract both cardiac and respiratory signals. X-ray projections with uniform time intervals can be obtained using different modes of microCT. In one mode, a series of X-ray projections is captured from a fixed angular position over time. In the other mode, one projection is captured per angular position as the X-ray source continuously rotates around the sample, resulting in a series of projections. This series of projections can capture diaphragm movements during the breathing process and, therefore, can be used for RR measurements. This type of scans generate signals that are uniformly sampled in the time-domain. The Signal Multiresolution Analyzer toolbox in Matlab offers user-friendly software for conducting FFT analysis on raw signals, significantly expediting the data processing when dealing with signals that are uniformly sampled in the time-domain. As a result, FFT analysis with this toolbox an optimal choice for processing uniform signals. Off note, in the first mode, since X-ray projections are obtained from a fixed-angle acquisition protocol these projections will not enable reconstruction into a 3D representation of the lungs, which is necessary for measuring other functional and morphological microCT-based biomarkers, such as aerated and non-aerated lung volumes and densities. In this study, we focused on a 4D time-resolved scanning protocol, which offers all applications microCT offers and does not restrict the imaging data to RR measurements or 3D data. The 4D scanning protocol used in this study was optimized by our group to be a physiologically radio-safe protocol for longitudinal animal studies (39). At each angular position during the scan, 9 projections are captured, corresponding to different phases of the breathing cycle. The results of the validation study demonstrated that the number of projections and exposure time provides a balanced temporal resolution, accurately capturing the RR of anesthetized animals while minimizing motion artifacts from respiration. Among all the signal processing techniques considered, MODWT algorithm exhibited the least accurate predictions for the ground-truth RRs. The low accuracy of this method is attributed to its dependency on a predefined frequency range of RRs for choosing the right signal component after the decomposition step, rather than based on the unique physiological characteristics of the individual mouse being investigated. While EMD algorithm employs intrinsic mode functions (IFMs) to capture local oscillations and non-stationary components within signals, which led to higher accuracy compared to MODWT, especially for RRs below 100 bpm. Nonetheless, similar to respiratory gating and MODWT, its accuracy sharply declined at RRs exceeding 100 bpm.

To validate our X-ray projection-derived RR, we applied the LS algorithm to microCT data from a NA-induced lung injury mouse model to longitudinally assess RR as a lung functional biomarker. The results showed a transient decrease in RR for the diseased mice which was paralleled by increase in the microCT-derived total lung volume and aerated lung volume, and its recovery. The transient reduction in RR, aligned with the established transient loss of lung function in the mouse model of NA-induced lung injury due to epithelial lung damage, highlights the sensitivity of RR as a lung function parameter. Notably, this reduction in RR aligns with increased total and aerated lung volumes, with no signs of inflammation or fibrosis in the lungs. Although lung function measures are biomarkers of lung injury, it remains unclear whether RR increases or decreases in response to different lung injuries. RR values measured with a barometric plethysmograph in bleomycin-induced mouse model of inflammation and fibrosis significantly dropped during the first two weeks, to recover during the third and fourth week after bleomycin treatment (31). The decrease in RR values after the bleomycin instillation was also paralleled by a decline in tidal volume (only after 3 days) and minute volume (days 3 and 7), attributed to the severe lung morphological alterations and loss of lung functional units. The authors concluded that a reduction in tidal volume accompanied by a decrease in RR is a normal physiological response aimed at minimizing the work of breathing. However, other longitudinal RR measurements in the same mouse model have shown the opposite effect on the animal’s RR, being a rapid and shallow breathing pattern (50, 51). This rapid, shallow breathing pattern, characterized by a higher respiratory rate and lower tidal volume, has also been reported in humans with idiopathic lung fibrosis (51–54). A similar rapid and shallow breathing pattern, marked by increase in RR and decrease in tidal volume, has been observed in other mouse model of lung injury, such as carrageenin-induced lung injury (55). These elevations in RRs were found to have a positive correlation with histopathology scores indicating alveolar damage and edema. These findings about RR in longitudinal animal studies, including our results in this study, clearly demonstrate the potential of RR as a valuable and translationally relevant lung functional biomarker. Although increasing our understanding of how the RR changes in response to different types of lung injury is warranted. The alterations in RR, depend on the lung disease and its stage of progression, are influenced by multiple factors such as ventilation distribution, airway resistance, and lung compliance. Pulmonary ventilation can be disrupted permanently or transiently by lung abnormalities, such as chronic obstructive pulmonary disease (COPD), which is directly linked to RR and tidal volume. To fully understand the mechanisms behind RR changes during lung injuries, further studies are needed to focus on respiratory mechanics, longitudinally track respiratory patterns, and link them to biomechanical properties such as airway resistance and lung compliance. Currently, longitudinal RR data should be interpreted cautiously in correlation with other longitudinal microCT-derived biomarkers, such as aerated and non-aerated lung volumes, as well as endpoint measurements such as histopathological sections. This pipeline delivering longitudinal X-ray-imaging derived RR data alongside full access to detailed microCT-derived biomarkers on lung pathology will greatly accelerate this workflow. All the more as microCT and this pipeline for RR extraction are fully generalizable across disease models as long as detectable intensity differences between the lower diseased lung and diaphragm remain. This workflow can be further accelerated through AI-based automation (56) and integration of signal processing steps (signal denoising, recognition and extraction) within image processing software packages.

Our proposed X-ray-based pipeline will have a direct impact on increasing our understanding of the role of RR as a lung functional biomarker for lung injuries by allowing RR to be linked to more relevant parameters of lung ventilation, such as minute ventilation. Minute ventilation, the product of RR and tidal volume, measures the amount of air entering the lungs per minute. Moreover, this approach enables non-invasive RR measurements at multiple time points without causing distress to animals, overcoming the limitations of current protocols for RR monitoring in preclinical settings. Developing such imaging-based techniques for pulmonary functional imaging will provide combined longitudinal and regional data on lung anatomy and physiology in a non-invasive manner, enhance sensitivity in detecting lung diseases at early stages and accurately assessing treatment outcomes. This approach also contributes to addressing the translational challenge in developing new therapeutics in preclinical settings. Despite extensive efforts to develop new therapeutic strategies, many promising drug candidates identified in animal models fail to replicate their beneficial effects in clinical trials. To better align outcome measures in preclinical settings and clinical trials, and to enhance insights into disease progression and drug responses, one solution lies in harmonizing methodologies. To achieve this, establishing a common language between animal and human studies through shared biomarkers, such as RR in lung research, is crucial. Imaging, by providing longitudinal data about anatomy and physiology in a non-invasive manner, can effectively fulfill this common role in better understanding the mechanisms behind pathologies in both animals and patients, as well as in developing more effective therapeutics to improve patient care.

## 5 Conclusion

We have introduced a new tool to extract RR as lung function biomarkers from longitudinal small animal microCT studies on lung diseases, obviating the need for using plethysmography techniques or camera-assisted cages. This new approach overcomes the necessity for additional experimental handlings, simplifies the experimental design and provides orthogonal data on lung disease burden and host response to inform biomarker interpretation. Here, we propose a non-invasive, X-ray-based pipeline for RR monitoring *in vivo* at multiple time points, offering simultaneous access to both anatomical and functional biomarkers in a single measurement. This approach eliminates the need for dedicated RR experiments, reducing costs and time while providing spatially resolved data on lung disease burden from microCT data. Moreover, imaging-based pipelines, by harmonizing methodologies in preclinical and clinical research, can help address the translational challenge in developing new therapeutics. Establishing a common language between animal and human studies through shared biomarkers, such as RR in lung research, is crucial. Imaging, as a non-invasive tool providing combined longitudinal anatomical and physiological data, can play this crucial role in understanding pathological mechanisms and advancing more effective therapeutics for improved patient care.

## Data Availability

The raw data supporting the conclusions of this article will be made available by the authors, without undue reservation.
